# A retrospective longitudinal assessment of artificial intelligence-assisted radiographic prediction of lower third molar eruption

**DOI:** 10.1038/s41598-024-51393-0

**Published:** 2024-01-10

**Authors:** Shivi Chopra, Myrthel Vranckx, Anna Ockerman, Peter Östgren, Carina Krüger-Weiner, Daniel Benchimol, Sohaib Shujaat, Reinhilde Jacobs

**Affiliations:** 1 Section of Oral Diagnostics and Surgery, Division of Diagnostics and Rehabilitation, Department of Dental Medicine, Karolinska Institutet, Alfred Nobels Allé 8, Huddinge, 141 53 Stockholm, Sweden; 2 OMFS-IMPATH Research Group, Department of Imaging and Pathology, Faculty of Medicine, University of Leuven, Leuven, Belgium; 3 Department of Oral and Maxillofacial Surgery, University Hospitals Leuven, Leuven, Belgium; 4 Department of Oral and Maxillofacial Radiology, Eastmaninstitutet, Folktandvården Stockholm Län AB, Stockholm, Sweden; 5 Department of Oral and Maxillofacial Surgery, Eastmaninstitutet, Folktandvården Stockholms Län AB, Stockholm, Sweden; 6 King Abdullah International Medical Research Center, Department of Maxillofacial Surgery and Diagnostic Sciences, College of Dentistry, King Saud bin Abdulaziz University for Health Sciences, Ministry of National Guard Health Affairs, Riyadh, Kingdom of Saudi Arabia

**Keywords:** Oral anatomy, Dental diseases, Oral diseases

## Abstract

Prediction of lower third molar eruption is crucial for its timely extraction. Therefore, the primary aim of this study was to investigate the prediction of lower third molar eruption and its uprighting with the assistance of an artificial intelligence (AI) tool. The secondary aim was identifying the incidence of fully erupted lower third molars with hygienic cleansability. In total, 771 patients having two panoramic radiographs were recruited, where the first radiograph was acquired at 8–15 years of age (T1) and the second acquisition was between 16 and 23 years (T2). The predictive model for third molar eruption could not be obtained as few teeth reached full eruption. However, uprighting model at T2 showed that in cases with sufficient retromolar space, an initial angulation of < 32° predicted uprighting. Full eruption was observed for 13.9% of the teeth, and only 1.7% showed hygienic cleansability. The predictions model of third molar uprighting could act as a valuable aid for guiding a clinician with the decision-making process of extracting third molars which fail to erupt in an upright fashion. In addition, a low incidence of fully erupted molars with hygienic cleansability suggest that a clinician might opt for prophylactic extraction.

## Introduction

The recent augmentation of artificial intelligence (AI) in oral radiology has allowed a more consistent and efficient approach towards classification, diagnostics and treatment planning^1–5^. The conventional time-consuming and observer dependent tasks are being constantly replaced by AI-based approaches which are able to either equal or surpass human accuracy^6^. The most remarkable progress related to AI in oral radiology has been the introduction of deep learning in the form of convolutional neural networks (CNNs)^7^. These networks mimic human cognition function in terms of learning and problem-solving and have been proven to be time-efficient and precise^8^. A task well suited for CNN in oral radiology is its assistance for the prediction of lower third molar eruption. However, the decision-making process on whether the tooth will erupt normally or stay impacted relies on regular clinical and radiological follow-up. An accurate prediction of an impacted tooth’s path might allow a clinician to perform a timely extraction at an early developmental stage, before it acquires a certain impacted position which might increase the risk of mandibular nerve injury and other complications^9^.

Previously, Vranckx et al.^10^ identified that third molar follicles with an initial angulation of greater than 27° relative to the second molar are predictive of a compromised eruption. In another study, the authors developed a CNN-based AI tool for assisting with the prediction process by allowing automated angulation measurements^11^. However, both studies were limited to the Belgian population and the timeframe between the two radiographs was narrow to allow for a precise prediction. Considering the aforementioned limitations, the rationale for conducting this study was to improve the generalizability of the AI tool and prediction model with a distinct population group having a broader age range. Furthermore, scarcity of evidence exists related to the incidence of third molars that can be effectively cleaned based on either clinical or radiological parameters^12^. This concept, known as ‘hygienic cleansability’, can be radiologically defined as fully erupted lower third molars at the level of second molar’s occlusal plane, having the marginal bone situated beneath the cementoenamel junction (CEJ) on the distal side. This positioning facilitates cleanliness while preventing pathological conditions, such as pericoronitis, periodontitis and caries. Clinically, fully erupted lower third molars that are functional, symptom-free, caries-free, positioned with a healthy periodontium, and not associated with other pathological conditions, are deemed hygienically cleansable and do not require extraction^12^. The documentation of such occurrences could enhance the decision-making process. If a population-based incidence of hygienic cleansability is low, it would imply that routine maintenance and periodic clinical and radiographic monitoring should be made mandatory for accommodating early preventive measures.

The primary aim of the present study was to predict the eruption and uprighting of lower third molar in a Swedish population group with a wide age gap between the acquired radiographs using a CNN-based AI tool, in an attempt to enhance the decision-making process related to its timely extraction. The secondary aim focused on identifying the incidence of fully erupted lower third molars based on radiological features that can be effectively cleaned, in order to assess whether patients can maintain adequate hygienic cleansability or are at an increased risk of developing a pathological condition that warrants extraction. The null hypothesis was that the AI-based model’s performance for assessing lower third molar eruption and uprighting prediction would be similar, and no difference would exist between the incidence of fully erupted third molars with and without hygienic cleansability.

## Methods

### Ethical declarations

This radiological retrospective longitudinal study was approved by the Swedish Ethical Review Authority (Dnr 2019-04736). All methods were performed in accordance with the relevant guidelines and regulations**.** Informed consent was waived by the Swedish Ethical Review Authority.

### Data collection

Panoramic radiographs of 10,921 patients having at least 2 radiographs were initially screened from the Electronic Healthcare system (T4 Practice Management Software; Carestream Dental; Altanta; GA; USA) of a Public Dental Service (Folktandvården Stockholm, Sweden). Radiographs and data including age and gender were extracted through Planmeca Romexis (Romexis 3.2.0; Planmeca; Helsinki; Finland). Data anonymization was achieved by removing the personal details of each patient and replacing them with a unique code number.

Patients who underwent two panoramic radiographs with good contrast, high image definition and without any distortion, artefacts or positioning errors that could negatively affect the measurements, and with a time difference of at least one year between both acquisitions were included, where the first radiograph was acquired at 8–15 years of age (T1) and the second acquisition was between 16 and 23 years (T2). These longitudinal radiographs were taken depending on the patient’s diagnostic or clinical needs, such as restorative treatment of permanent teeth, dental screening, orthodontic alignment and restoration or extraction of deciduous teeth. In addition, inclusion criteria consisted of fully erupted lower dentition with the exception of third molars at T1 time-point and fully erupted lower dentition with either unerupted or fully erupted lower third molars at T2 time-point. Exclusion criteria were patients with supernumerary teeth and odontomas, previous history of maxillofacial trauma or reconstructive surgery, orthodontic extraction therapy and presence of craniofacial anomalies such as cleft lip and/or palate, hemifacial microsomia, craniosynostosis and other syndromic diseases.

The selection of radiographs based on the inclusion criteria was performed using consecutive non-probability sampling technique by a single dentist having an experience of over 6 years, followed by reconfirmation by an oral radiologist. If a consensus could not be reached, a senior consultant oral and maxillofacial radiologist was consulted. All image data were anonymized prior to analysis An a priori power analysis was conducted using G*power 3.1, to determine the minimum sample size required for the study. The analysis was based on a mean angular difference of 2.1 ± 13.8°, with 80% power at a significance level of 5%, in accordance with a previous study^10^. The minimum sample size was calculated to be 1072 lower third molars (536 patients).

### Recorded variables

The recorded parameters for predicting third molar eruption and its uprighting included, third molar’s developmental stage, angulation difference between second and third molars, third molar eruption level and available retromolar space.

**Figure 1:**
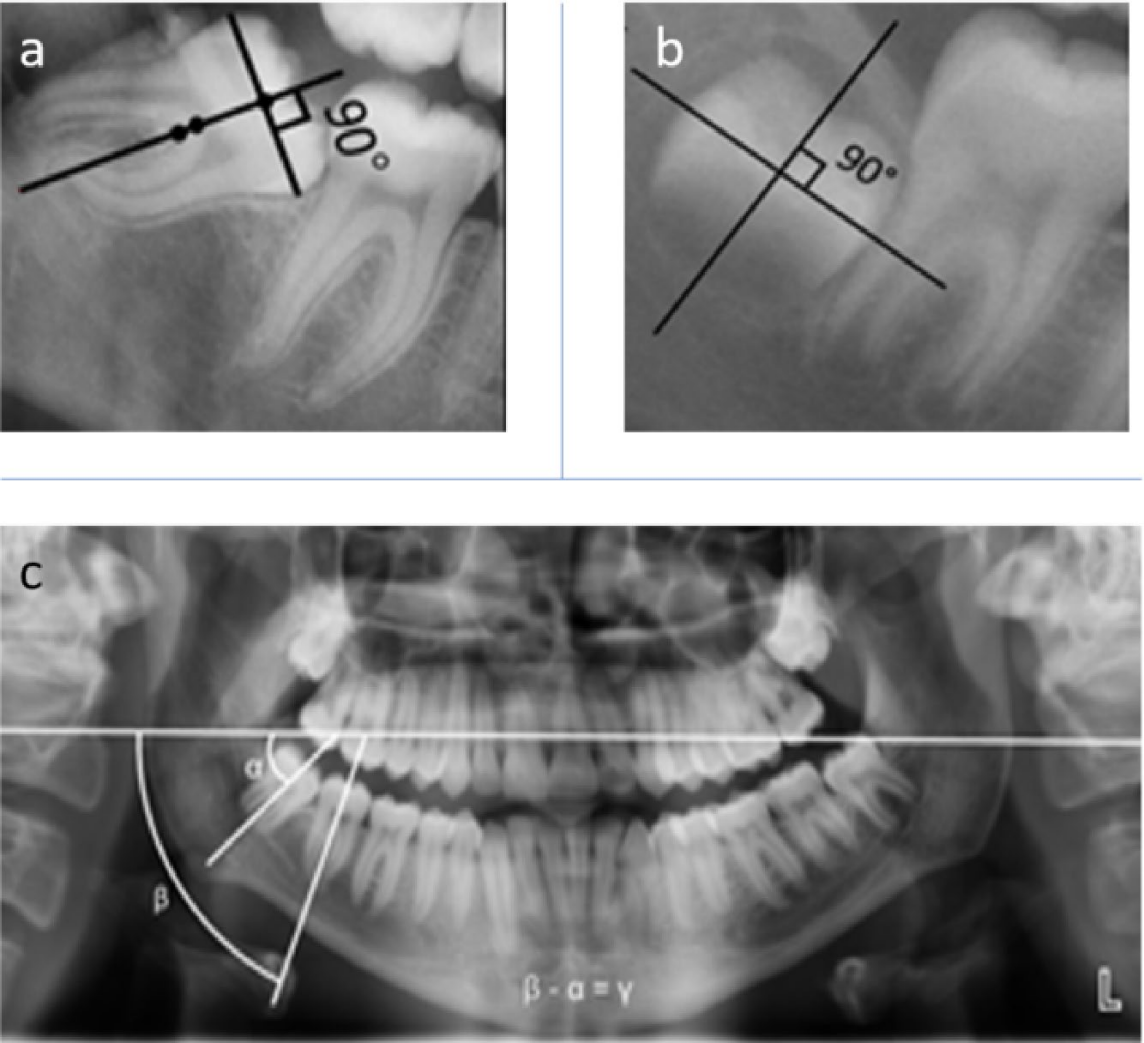
Calculation of lower third molar angulation. (**a**) Calculation of angulation on molars with fully formed roots where a line was drawn at region of largest coronal diameter and angulation line was drawn from most apical part of pulp chamber or most coronal part of bifurcation area; (**b**) calculation of angulation on molar with incompletely formed roots where the crown was divided into two equal halves, midpoint of widest diameter was taken and an inclination line was drawn perpendicularly (90°); (**c**) third molar angle defined based on angular difference (γ) between second (β) and third molar (α), represented by β − α = γ.

Firstly, the level of third molar development was categorized as either having incompletely or fully formed roots. Thereafter, the panoramic radiographs at both T1 and T2 time-points were imported to a previously developed and validated AI tool for performing automated molars segmentation and angulation measurements. The angulation of third molars with fully formed roots were automatically measured by dividing the crown into two equal halves and then taking the midpoint of the widest diameter of the crown. The inclination line was then drawn perpendicularly (90°) against this line. In cases with incompletely developed roots the angulations were measured automatically by drawing a line at the region of largest diameter of the crown and the angulation line was made from the most apical part of the pulp chamber or the most coronal part of the bifurcation area. Angulations of the second and third lower molar were assessed relative to the horizontal plane of the radiograph. Finally, the AI tool provided the final angle of the third molar by assessing the angular difference between second and third molar (γ) (Fig. 1a–c). If the angle of third molar was equal to or less than 15° at T2 then it was classified as uprighted.

**Figure 2:**
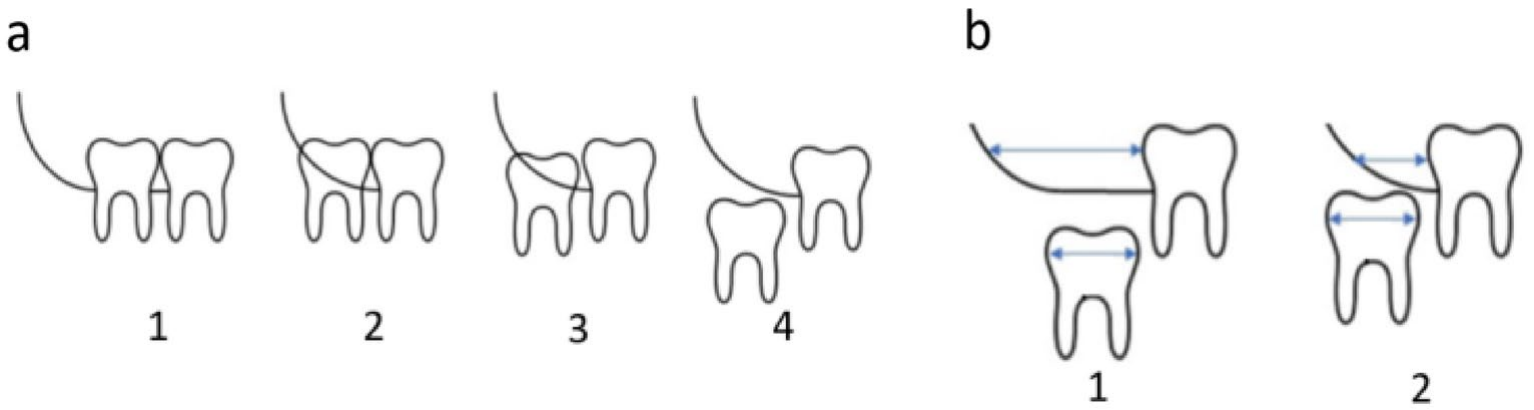
Eruption levels of lower third molar and retromolar space. (**a**) 1: fully erupted with hygienic cleansability where third molar is at level of 2nd molar’s occlusal plane with marginal bone situated beneath the CEJ at distal side, 2: fully erupted without hygienic cleansability where third molar is at level of 2nd molar’s occlusal plane with marginal bone above CEJ at distal side, 3: partially erupted with height of third molar contour above level of surrounding alveolar bone, 4: unerupted with third molar completely encased in bone; (**b**) available retromolar space, where 1: sufficient space, widest mesiodistal crown width of third molar fits available space measured between distal side of second molar till anterior border of ramus, 2: insufficient space, widest mesiodistal crown width of third molar does not fit available space.

The level of third molar eruption was further classified into four categories as follows; fully erupted with hygienic cleansability: erupted up to the level of occlusal plane of the second molar with the marginal bone situated beneath the CEJ at distal side suggesting hygienic cleansability, fully erupted without hygienic cleansability: erupted up to the level of occlusal plane of the second molar with the marginal bone situated above the CEJ at distal side without the ability to properly maintain oral hygiene, partially erupted: the height of tooth’s contour is above the level of surrounding alveolar bone, non-erupted: bony impaction, the tooth is completely encased in bone (Fig. 2a).

Following third molar assessment, the available retromolar space was graded manually by an observer to assess the eruption space in accordance with a modified protocol described by Hattab and Alhaija. The criteria consisted of the following, sufficient space: widest mesiodistal crown width of the third molar fits the available space (measured between the distal side of second molar till anterior border of ramus) and reduced or insufficient space: available space is less than the third molar’s mesiodistal crown width (Fig. 2b).

### Statistical analysis

Data were analyzed with S-plus 8.0 for Linux (S-plus 8.0; Tibco software; Palo Alto; CA). Descriptive statistics were applied to assess the incidence of lower third molar’s full eruption with and without hygienic cleansability and the impact of retromolar space on hygienic cleansability. A general linear mixed model with stepwise effect selection and fitting via the logit link was used to make prediction models for both third molar eruption and uprighting between T1 and T2. The decision for using this model was based on its ability to handle binary data. Moreover, a stepwise effect selection was added to observe which combination of the measured parameters made the strongest prediction model based on the Type III p-value. A threshold of 0.1 was used to put variables into the model and a p-value of 0.2 was used to leave variables out. Instead of relying on average combined data, the data from both left and right side from all the patients was used separately and the patient was modelled as a random factor. Moreover, Receiver operating characteristic (ROC) curves were generated for third molar eruption prediction models based on training and validation datasets.

## Results

Following the eligibility criteria, 771 patients (391 males, 380 females) with each patient having two panoramic radiographs were selected, accounting for a total of 1542 lower third molars at T1 and T2 time-points. The average time-interval between T1 and T2 was 4.5 ± 2.2 years, where the average age of patients at T1 time-point was 14.1 ± 1.0 years and 18.5 ± 2.0 at T2. Of the total third molar sample, 13.9% (214/1542) showed full eruption at T2, while 1.7% (26/1542) observed hygienic cleansability. Based on available retromolar space, 39% (16/41) of cases with sufficient space observed full eruption with hygienic cleansability, whereas only 0.06% (10/1501) of the patients were able to properly maintain oral hygiene with insufficient retromolar space.

The stepwise effect selection showed that variables that most accurately predicted both third molar eruption (with and without hygienic cleansability) and uprighting were its angulation combined with retromolar space. As very few third molars reached full eruption, a clinically applicable model for full eruption could not be obtained based on third molar angulation at T1 combined with retromolar space.

**Figure 3:**
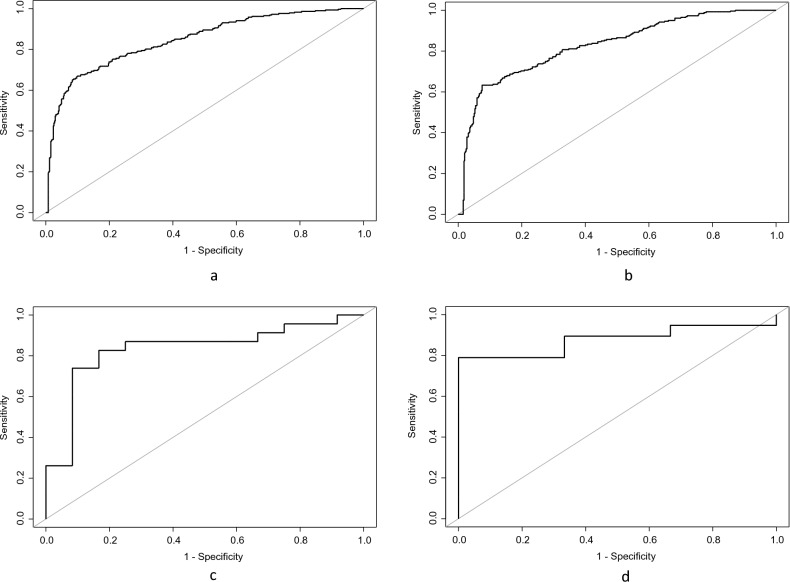
Receiver operating characteristic (ROC) curves of third molar eruption prediction. (**a**) uprighting with reduced or insufficient retromolar space on training dataset; (**b**) uprighting with reduced or insufficient retromolar space on validation dataset; (**c**) uprighting with sufficient retromolar space on training dataset; (**d**) uprighting with sufficient retromolar space on validation dataset.

Prediction models for third molar uprighting at T2 based on third molar angulation and available retromolar space showed that when retromolar space was reduced or insufficient, an initial angle of γ < 21° at T1 predicted uprighting at T2. The positive predictive value (PPV) of the model was 67%, whereas the negative predictive value (NPV) was 85%. In terms of sensitivity and specificity, the model scored 75% and 79% respectively (Fig. 3a). With the model running on a validation dataset, it achieved a PPV and sensitivity of 67%, and NPV and specificity of 82% (Fig. 3b). When retromolar space was sufficient, an initial angle of γ < 32° at T1 predicted uprighting at T2. The PPV of this model was 91%, while the NPV was 71%. Furthermore, the model demonstrated a sensitivity and specificity of 83%. (Fig. 3c). Based on the validation set, the model demonstrated a PPV of 100%, NPV of 38%, along with a sensitivity and specificity of 74% and 100%, respectively. (Fig. 3d).

## Discussion

The null hypothesis was rejected as the predictive model for third molar eruption could not be established and a low incidence of patients existed having fully erupted teeth with radiological features suggesting hygienic cleansability.

The prediction of third molar eruption is an area of great interest for clinicians since it can improve the current standard of patient care. Also, only a few studies have attempted to predict the lower third molar eruption over time^13,14^. The main limitation associated with these studies has either been the lack of longitudinal radiographic datasets or a small sample size owing to the removal of third molar during follow-up visits^15,16^. In addition, there is a paucity of evidence concerning the incidence of fully erupted third molars exhibiting radiological characteristics indicative of hygienic cleansability, a crucial determinant in the extraction decision-making process. This can be achieved by ensuring that the tooth is unobstructed by any soft or hard tissue, as most pathological diseases such as pericoronitis, periodontitis and caries are often associated with partially impacted lower third molars^17,18^. Therefore, the following study was conducted to predict the third molar eruption and uprighting based on different variables with the assistance of an AI tool and to report on the incidence of erupted third molars having radiological features suggestive of hygienic cleansability.

The present study used an automated AI tool for measuring the third molar angulation, which has been technically and clinically validated in a prior study^11^. Hence, no further intra- or inter-examiner assessment was required. Gender discrimination was not taken into consideration as it has been previously reported that gender has no significant impact on third molar eruption^19^. Several factors have been reported in literature which influence the probability of mandibular third molar eruption^20,21^. Amongst these factors, the availability of retromolar space is one of the most prime factors^14^, which was also in accordance with our findings where patients with insufficient retromolar space were prone to a higher risk of impaction^22^.

An attempt was made to draw a prediction model based on third molar angulations at T1 combined with available retromolar space for full eruption. However, the limited sample size of fully erupted third molars precluded the development of such a model. This limitation can be attributed to the fact that the T1 radiographs were from patients aged between 8 and 15 years, a demographic that typically presents with orthodontic issues necessitating clinical examination supplemented with panoramic radiography, a common practice in orthodontics^23^. This may have introduced a selection bias, as the majority of these patients could have crowded teeth due to a smaller mandible and higher risk of impacted mandibular third molars compared to the general population^19,24,25^. Another potential factor contributing to the elevated occurrence of unerupted mandibular third molars could be the average age of the patients at the second time point (T2), which was 18.5 years. It has been observed that teeth impacted at the age of 18 years, may have a probability ranging from 30 to 50% of eventually fully erupting, provided they are not impacted in a horizontal orientation^21^. Nevertheless, it was still feasible to predict the uprighting of the third molar, with full eruption occurring at angular cut-off angular values of < 21.28°–31.54° at T1, which was consistent with a prior study^10^. Given that the average age at T2 was 18.5 years, and considering that many third molars tend to fully erupt later, upright angulation was employed as a marker for subsequent eruption. The predictions models for uprighting were considered strong, reaching values of around 75% and the results also proved to be robust and sustainable in the validation datasets. Hence, suggesting its clinical applicability for predicting third molar uprighting. An addition of larger sample with a higher age at T2 might improve the model’s prediction performance, which should be investigated in future studies. Previously, Vranckx et al.^10^ studied the relationship between third molar angulation and eruption and they reported that the recruited patients in their sample might have been too young at T2 to draw relevant conclusions related to the prediction. In contrast, the sample in present study included patients with a higher age at T2. Nevertheless, as the data of fully erupted molars was still scarce, more longitudinal data collection of patients having fully erupted teeth at T2 should be beneficial in improving the prediction workflow.

The findings suggested a low incidence of patients having fully erupted teeth with hygienic cleansability, which was in accordance with prior studies where the risk of partial impaction was higher with a high degree of pericoronitis^17,18^. As the life-expectancy of Swedish population has been documented to increase by each generation^27^ with a higher number of teeth being preserved^28^, the probability of pathology-free partially erupted lower third molars during one’s lifetime is limited. Hence, eruption prediction could allow to prophylactically extract these teeth with a less risk of complications^29,30^.

The main strengths of the study were the application of an AI tool for assisting with the angulation measurement which increased the time-efficiency of the evaluation, while also helping to optimize the tool’s performance and its generalizability with the inclusion of heterogenous dataset at different time-intervals. Although AI assisted in only automatically calculating the third molar’s angulation, further studies are warranted to also include retromolar space assessment and other variables in an attempt to make the entire process of eruption prediction automated. It would be of valuable interest to iterate the proposed research set-up to a sample of higher age groups at T2, preferably at the age of 21–23 years old^26^, which could improve the prediction model. Third molars might still fail to erupt even if all radiographic indicators are favorable, thereby, it is necessary to complement radiographic information with patient genomics which might shed some light onto the eruption physiology.

The study had some limitations. Firstly, the eruption of the lower third molars was not clinically verified and hygienic cleansability was also only assessed radiologically. These findings should be interpreted with caution as the impact of soft tissue and other clinical parameters such as plaque index, bleeding, pericoronitis episodes were not investigated. Hence, it is recommended to perform further studies by focusing on a combination of both radiological and clinical parameters. Secondly, the initial selection of panoramic images was performed by one evaluator, which could have contributed towards selection bias. Finally, patients with a previous history of orthodontic teeth alignment therapy were also included in the study where alignment could have influenced the eruption path^31^. Thereby, it is important to train the model based on different factors to improve its performance.

## Conclusions

Although it was not possible to predict the eruption of lower third molars, a strong prediction model was developed for predicting molar uprighting. This could help clinicians improve the decision-making process concerning extraction. Moreover, the likelihood of having lower third molars without any pathology throughout one’s life may be limited due to the low incidence of fully erupted third molars with radiological features that suggest hygienic cleansability.

## Data Availability

The dataset analyzed during the current study are available from the corresponding author on reasonable request.
