# Author Correction: A retrospective longitudinal assessment of artificial intelligence-assisted radiographic prediction of lower third molar eruption

**DOI:** 10.1038/s41598-024-57144-5

**Published:** 2024-03-19

**Authors:** Shivi Chopra, Myrthel Vranckx, Anna Ockerman, Peter Östgren, Carina Krüger‑Weiner, Daniel Benchimol, Sohaib Shujaat, Reinhilde Jacobs

**Affiliations:** 1 Section of Oral Diagnostics and Surgery, Division of Diagnostics and Rehabilitation, Department of Dental Medicine, Karolinska Institutet, Alfred Nobels Allé 8, Huddinge, 141 53 Stockholm, Sweden; 2 OMFS-IMPATH Research Group, Department of Imaging and Pathology, Faculty of Medicine, University of Leuven, Leuven, Belgium; 3 Department of Oral and Maxillofacial Surgery, University Hospitals Leuven, Leuven, Belgium; 4 Department of Oral and Maxillofacial Radiology, Eastmaninstitutet, Folktandvården Stockholm Län AB, Stockholm, Sweden; 5 Department of Oral and Maxillofacial Surgery, Eastmaninstitutet, Folktandvården Stockholms Län AB, Stockholm, Sweden; 6 King Abdullah International Medical Research Center, Department of Maxillofacial Surgery and Diagnostic Sciences, College of Dentistry, King Saud bin Abdulaziz University for Health Sciences, Ministry of National Guard Health Affairs, Riyadh, Kingdom of Saudi Arabia

Correction to: *Scientific Reports* 10.1038/s41598-024-51393-0, published online 10 January 2024

The original version of this Article omitted an affiliation for Sohaib Shujaat. The correct affiliations are listed below.

OMFS-IMPATH Research Group, Department of Imaging and Pathology, Faculty of Medicine, University of Leuven, Leuven, Belgium

Department of Oral and Maxillofacial Surgery, University Hospitals Leuven, Leuven, Belgium

King Abdullah International Medical Research Center, Department of Maxillofacial Surgery and Diagnostic Sciences, College of Dentistry, King Saud bin Abdulaziz University for Health Sciences, Ministry of National Guard Health Affairs, Riyadh, Kingdom of Saudi Arabia

The original Article has been corrected.

